# Optimization of HEK 293 cell growth by addition of non-animal derived components using design of experiments

**DOI:** 10.1186/1753-6561-5-S8-P126

**Published:** 2011-11-22

**Authors:** Laura Cervera, Sonia Gutiérrez, Francesc Gòdia, María M Segura

**Affiliations:** 1 Departament d'Enginyeria Química, Universitat Autònoma de Barcelona, Bellaterra, Barcelona, 08193, Spain

## Background

Mammalian cells are a widely used expression platform for the production of recombinant therapeutic proteins or viral particle-based vaccines since they typically perform appropriate protein post-translational modifications and authentic viral particle assembly. Of the available mammalian cells, HEK 293 is one of the most industrially relevant cell lines because it is cGMP compliant and is able to grow in suspension in a variety of commercial serum-free media. Of note, production of human therapeutics in mammalian cell culture has become more and more stringent in past recent years and not only demands serum-free but also animal-component free production conditions to ensure safety. This work is part of a project aimed to optimize HEK 293 cell growth by addition of non-animal derived components to serum-free and protein-free media through design of experiments (DoE) in order to maximize productivity of a recombinant VLP vaccine by PEI-mediated transient transfection.

## Materials and methods

The cell line used in this work is a serum-free suspension-adapted HEK 293 cell line from a cGMP master cell bank from the Biotechnology Institute of the National Research Council in Montreal, Canada. Cells were maintained in exponential growth in 125-mL disposable polycarbonate Erlenmeyer flasks shaken at 110 rpm using an orbital shaker placed in an incubator with a 37°C, humidified, 5% CO_2_ atmosphere. Cell count and viability were determined using trypan blue and a microscope counting chamber.

## Results

**Figure 1:**
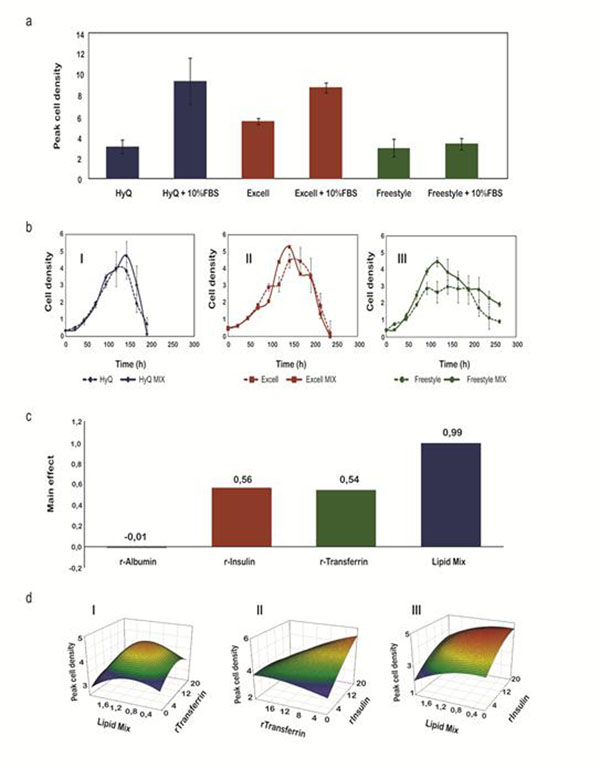
Optimization of HEK 293 cell growth a) The effect of 10% FBS supplementation in commercially av ailable serum-free media, b) Growth kinetics of HEK 293 cells in 3 different serum-free protein-free formulations (bI, bII and bIII) in the presence or absence of a pre-defined mixture of supplements, c) Effect of recombinant proteins or synthetic lipid mix on HEK 293 cell growth, d) Response surface graphs. HEK 293 peak cell density as a function of the concentrations of Lipid Mix (X) vs. r-Transferrin (mg/L) (dI), r-Transferrin (mg/L) vs. r-Insulin (mg/L) (dII) and Lipid Mix (X) vs. r-Insulin (mg/L) (dIII) based on Box-Behnken experimental results. Cell density values presented are in millions of cells/mL and represent mean ± SD (n=3).

We have analyzed the kinetics of HEK 293 cell growth in HyQ SFM4 Transfx293 from HyClone Thermo Scientific (Logan, UT, USA). The cells grow to a maximum concentration of ~ 3×10^6^ cells/ml with over 90% viability and show an average doubling time of 24 h. In addition, HEK 293 cell growth was assessed in two other commercial serum-free culture media, namely ExCell 293 from SAFC Biosciences (Hampshire, UK) and Freestyle 293 from Invitrogen (Carlsbad, CA, USA) both compatible with PEI-mediated transient transfection , showing similar results (Figure 1a). The effect of foetal bovine sera (FBS) in these serum-free culture media was also evaluated. Cells can triplicate their maximum cell densities in the presence of FBS (i.e. they reach 9,3×10^6^ cells/ml in HyQ SFM4 Transfx293 medium + 10% FBS).

Due to the important effect of serum on HEK 293 cell growth, we decided to evaluate the effect of non-animal derived serum components on cell growth in attempt to improve cell densities while keeping animal-origin free production conditions. For these studies, a pre-defined mixture of supplements composed of r-albumin (1 g/L), r-insulin (10 mg/L), r-transferrin (10 mg/L) from Merck Millipore (Kankakee, IL, USA), and an *in-house* developed animal-component free lipid mix (1X) composed of synthetic cholesterol (SyntheChol^®^, Sigma-Aldrich, Steinheim, Germany), fatty acids (F7050, SAFC Biosciences), tocopherol (T1157, Sigma) and emulsifying agents (PS80, Sigma) at concentrations recommended in the literature [1] were used. HEK 293 cell density is improved in the presence of the mix, but only in Freestyle medium a significant difference is observed (Figure 1b). This medium was selected for further optimization by DoE.

Screening of supplements with significant effect on HEK 293 cell growth was performed using a Placket-Burman experimental design [2]. Two levels of concentrations were assigned to each variable: a low one with no additives and a high one based on the typically recommended values mentioned above. Using this strategy, we were able to determine in 12 experimental runs (performed in duplicate) that r-insulin, r-transferrin and an *in-house* developed lipid mix positively affect HEK 293 cell growth in serum-free media formulations, whereas r-albumin showed no significant effect (Figure 1c).

Optimal concentrations for each supplement showing a significant effect on HEK 293 cell growth were defined based on a Box-Behnken experimental design [3]. Three levels of concentrations for each variable were selected (Table 1). Using this experimental design, we were able to define in 15 experimental runs (performed in duplicate) a model that accurately predicts HEK 293 cell concentrations in the presence of different concentrations of r-insulin (r-Ins), r-transferrin (r-Trans) and lipid mix (LipMix) (Table 1).

**Table 1:** Box-Behnken results. ^a^ Three levels of concentrations for each variable including a maximum (1), a minimum (-1) and a center point (0) were used. Values shown in parenthesis are concentrations employed for r-transferrin and r-insulin (mg/mL) and lipid mix (based on Synthechol^®^ concentrations provided in X). ^b^ Cell density values presented are mean of duplicate runs in million cells/mL.

EXP N°	r-Insulin^a^	r-Transferrin^a^	Lipid Mix^a^	Max Cell density^b^	
	
				Experimental	According to model
1	-1 (1)	-1 (1)	0 (1)	3,1	2,8
2	1 (20)	-1 (1)	0 (1)	5,5	5,4
3	-1(1)	1 (20)	0 (1)	3,7	3,8
4	1 (20)	1 (20)	0 (1)	3,3	3,7
5	-1 (1)	0 (10)	-1 (0.1)	3,1	3,1
6	1 (20)	0 (10)	-1 (0.1)	4,6	4,4
7	-1 (1)	0 (10)	1 (2)	2,0	2,3
8	1 (20)	0 (10)	1 (2)	3,6	3,6
9	0 (10)	-1 (1)	-1 (0.1)	3,2	3,6
10	0 (10)	1 (20)	-1 (0.1)	3,5	3,4
11	0 (10)	-1 (1)	1 (2)	2,8	2,9
12	0 (10)	1 (20)	1 (2)	2,8	2,4
13	0 (10)	0 (10)	0 (1)	4,9	4,5
13	0 (10)	0 (10)	0 (1)	4,9	4,5
13	0 (10)	0 (10)	0 (1)	3,8	4,5

The Box-Behnken model equation was**:**

Cell density (10^6^ cells/mL) = 4,53 + 0,63 × r-Ins - 0,17 × r-Trans - 0,41 × LipMix - 0,68 × r-Ins × r-Trans + 0,02 × r-Ins × LipMix - 0,07 × rTrans × LipMix - 0,18 × r-Ins^2^ - 0,45 × r-Trans^2^ - 1,02 × LipMix^2^

Optimal concentrations for each supplement in HEK 293 cell culture medium were defined based on this model to be 19,8 mg/L of r-insulin, 1,6 mg/L of r-transferrin and 0,9X for the lipid mix. These results can be inferred from response surface graphs (Figure 1d). Finally, the model was validated experimentally. For this purpose, the growth kinetics of HEK 293 cells in the optimized cell culture medium was analyzed. In the presence of the suitable combination/concentrations of supplements, HEK 293 cells reached a maximum cell density of 5,4x10^6^ cells/mL (n=3), same value as predicted using the Box-Behnken model, as opposed to the unsupplemented Freestyle medium that supported cell growth up 3x10^6^ cells/mL (n=3) (Figure 1a & 1bIII).

## Conclusions

Results have shown that by adding a mixture of animal-free supplements to serum-free culture medium, it is possible to reach high cell densities comparable to those attained in the presence of FBS while avoiding the problems derived from its use.
